# TLR2, but Not TLR4, Is Required for Effective Host Defence against *Chlamydia* Respiratory Tract Infection in Early Life

**DOI:** 10.1371/journal.pone.0039460

**Published:** 2012-06-19

**Authors:** Emma L. Beckett, Simon Phipps, Malcolm R. Starkey, Jay C. Horvat, Kenneth W. Beagley, Paul S. Foster, Philip M. Hansbro

**Affiliations:** 1 Centre for Asthma and Respiratory Disease, The University of Newcastle, and Hunter Medical Research Institute, Newcastle, New South Wales, Australia; 2 School of Biomedical Sciences and Australian Infectious Diseases Research Centre, The University of Queensland, Brisbane, Queensland, Australia; 3 Institute of Health and Biomedical Innovation, Queensland University of Technology, Brisbane, Queensland, Australia; Albany Medical College, United States of America

## Abstract

*Chlamydia pneumoniae* commonly causes respiratory tract infections in children, and epidemiological investigations strongly link infection to the pathogenesis of asthma. The immune system in early life is immature and may not respond appropriately to pathogens. Toll-like receptor (TLR)2 and 4 are regarded as the primary pattern recognition receptors that sense bacteria, however their contribution to innate and adaptive immunity in early life remains poorly defined. We investigated the role of TLR2 and 4 in the induction of immune responses to *Chlamydia muridarum* respiratory infection, in neonatal wild-type (Wt) or TLR2-deficient (^−/−^), 4^−/−^ or 2/4^−/−^ BALB/c mice. Wt mice had moderate disease and infection. TLR2^−/−^ mice had more severe disease and more intense and prolonged infection compared to other groups. TLR4^−/−^ mice were asymptomatic. TLR2/4^−/−^ mice had severe early disease and persistent infection, which resolved thereafter consistent with the absence of symptoms in TLR4^−/−^ mice. Wt mice mounted robust innate and adaptive responses with an influx of natural killer (NK) cells, neutrophils, myeloid (mDCs) and plasmacytoid (pDCs) dendritic cells, and activated CD4^+^ and CD8^+^ T-cells into the lungs. Wt mice also had effective production of interferon (IFN)γ in the lymph nodes and lung, and proliferation of lymph node T-cells. TLR2^−/−^ mice had more intense and persistent innate (particularly neutrophil) and adaptive cell responses and IL-17 expression in the lung, however IFNγ responses and T-cell proliferation were reduced. TLR2/4^−/−^ mice had reduced innate and adaptive responses. Most importantly, neutrophil phagocytosis was impaired in the absence of TLR2. Thus, TLR2 expression, particularly on neutrophils, is required for effective control of *Chlamydia* respiratory infection in early life. Loss of control of infection leads to enhanced but ineffective TLR4-mediated inflammatory responses that prolong disease symptoms. This indicates that TLR2 agonists may be beneficial in the treatment of early life *Chlamydia* infections and associated diseases.

## Introduction

*Chlamydiae* are obligate intracellular bacteria, and have an atypical Gram-negative cell wall [1]. *C. pneumoniae* commonly infects the respiratory tract of children causing upper and lower respiratory problems [2], and is increasingly associated with severe childhood asthma [1], [3], [4]. These infections are probably significantly under-reported due to a lack of investigation and standardized diagnostic techniques [5]. Despite the high prevalence in children few studies have investigated the inflammatory and immune responses to *Chlamydia* respiratory infection in this age group [1]. We have previously used experimental models to demonstrate that *Chlamydia* respiratory infections in early life may increase the severity of asthma by inducing mixed T-cell responses, enhancing IL-13 and -17 expression and mucus hypersecretion in the lung, and altering lung structure and function [6]–[11]. However, the early innate responses that protect against infection in early life have not been elucidated.

The respiratory tract is the first point of contact for inhaled pathogens and has a complex network of innate and adaptive immune responses that protect against infection. Innate responses are initiated by the recognition of pathogen-associated molecular patterns by pattern recognition receptors (PRRs). TLRs are an important family of PRRs, which are expressed on epithelial cells, neutrophils, macrophages and DCs, and are critical for the induction of innate and adaptive immunity [12]. TLR activation initiates downstream signalling through myeloid differentiation factor 88 (MyD88), which induces transcription factors and the expression of inflammatory and immune genes and responses. *Chlamydia* predominantly induces TLR2 but also TLR4 responses, through the recognition of cell wall components (e.g. LPS and potentially peptidoglycan) [13]–[15] and heat shock proteins [16]. The absence of TLR2 and 4 in adult mice has no impact on mortality rate, inflammatory profile or *C. pneumoniae* clearance [17]. However, these results are controversial since it has also been demonstrated that TLR2 is crucial for neutrophil responses to *C. pneumoniae* infection [18]. Recent studies, also in adult mice, show that TLR2 is required to control inflammation but not *C. muridarum* respiratory infection (replication) [19]. The fact that both TLR2 and 4 are involved in responses to *Chlamydia* and both signal through MyD88 suggests that there may be some molecular redundancy that may be elucidated using TLR2/4^−/−^ mice.

Neutrophils and macrophages are the dominant innate immune cells in the lung and bind to pathogens through TLRs. This induces phagocytosis and destruction *via* the release of anti-microbial factors including nitric oxide, superoxide anions and hydrogen peroxide. Macrophages are resident in the airways, and upon encounter with pathogens release chemotactic factors that recruit neutrophils to the site of infection [20]. In Wt mice, respiratory infection with the natural mouse pathogen *C. muridarum* results in a pronounced neutrophil infiltration during the acute response, which corresponds with the peak of infection [6]. Activated macrophages and DCs recruit NK cells, which recognise and kill cells infected with intracellular bacteria through cytotoxicity and secretion of IFN**γ** [21]. Innate responses also induce subsequent adaptive immunity and acquired immunological memory. DCs play particularly crucial roles by processing and presenting antigens and activating adaptive responses from T- and B-cells [12]. DCs may be classified into mDCs and pDCs on the basis of differing phenotypic markers, cytokine production and origin [22]. mDCs and pDCs are considered more important in bacterial and viral infections, respectively, however the roles of DC subtypes in *Chlamydia* infections remain to be elucidated.

Murine studies have demonstrated that CD4^+^ and CD8^+^ T-cells are essential for protection against *Chlamydia* respiratory infection [23], whereas humoral immunity does not appear to be important [24]. CD4+ T-helper type 1 (Th1) cells secrete the cytokines IL-2, IFN**γ** and TNF, induce cell-mediated immunity by activating macrophages and cytotoxic T-cells, and are crucial in the clearance of intracellular pathogens [6], [11]. IFN**γ** released from innate immune cells and T-cells is particularly important in the clearance of *Chlamydia* from the lung [23], [25], [26].

The age of first exposure to infectious microorganisms shapes the phenotype of immune responses later in life [1], [6], [27], [28]. *Chlamydia* infection in neonatal mice induces the same infection and inflammatory profiles as in adults [6], [8], however, the nature and effectiveness of the subsequent immune response in early life remains unknown. Although the clearance of *Chlamydia* infections requires an adequate Th1 response, the neonatal immune system is primed towards a Th2 bias [1], the induction of Th1 responses is weak and unstable [7], [29], and lymphocyte and macrophage activity is impaired [30]. Thus neonates may be less capable of clearing a *Chlamydia* infection that results in more severe tissue damage and maladaptive immune responses that increase the risk of subsequent disease pathologies in later life.

In this study we investigated the inflammatory and immune responses to respiratory *Chlamydia* infection in early life using *C. muridarum*. The roles of TLRs in these responses were clarified using TLR2^−/−^, 4^−/−^ and 2/4^−/−^ mice.

## Results

### Absence of TLR2 reduces weight gain and increases clinical score during *Chlamydia* respiratory infection in early life

We first assessed the impact of the absence of TLR2 and 4 on neonatal growth and clinical features during infection. Sham or *C. muridarum* respiratory tract infection was induced in neonatal Wt, TLR2^−/−^, 4^−/−^ and 2/4^−/−^ mice and weight gain and clinical score assessed over a 0–14 day time course. Pups receiving sham inoculation had a linear increase in percentage of weight gained over time, and no differences were observed between Wt and TLR^−/−^ mice (Figure S1A). No clinical symptoms were observed.

**Figure 1:**
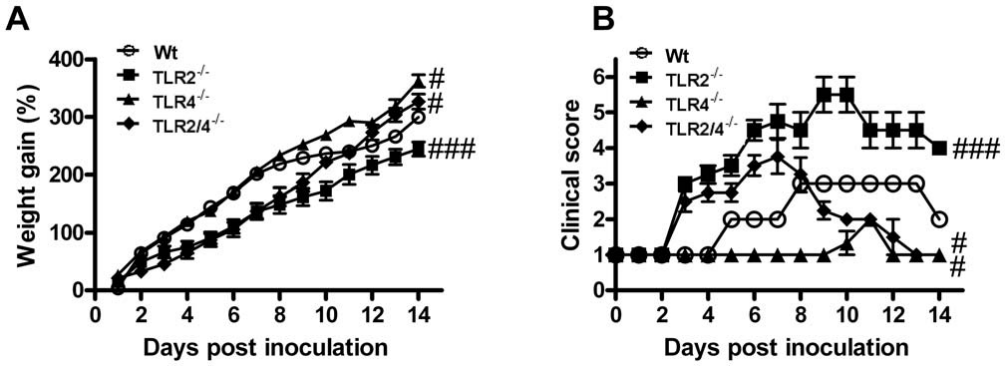
Absence of TLR2 reduces weight gain and increases clinical score during *Chlamydia* respiratory infection in early life. Wild type (Wt) and TLR^−/−^ pups were sham inoculated or infected. (A) Mice were weighed individually each day and the percentage weight gain calculated daily relative to the average weight of the litter prior to inoculation. n = 10–12 pups/group (5–6 pups/litter). (B) Clinical scores were also determined based on symptoms observed in litters daily as described in Table S1. n = 2–4 litters. Results are presented as means ± SEM. # denotes significant difference between infected Wt and TLR^−/−^ groups. # p<0.05, ### p<0.001 for the whole curves.

Following inoculation with *C. muridarum*, Wt pups had reduced weight gain between 11–14 days post-inoculation (dpi), compared to un-infected controls (Figure S1B). The absence of TLR2 resulted in a further reduced weight gain from 5 dpi until the end of the time course, compared to infected Wt mice (Figure 1A). By contrast TLR4^−/−^ mice showed no growth retardation and actually had increased weight gain compared to infected Wt mice from 10 dpi. Absence of both TLR2 and 4 resulted in growth reduction between 5–8 dpi but increased weight gain 12–14 dpi, compared to Wt. Thus, TLR2 and 4 double deficiency had the same effects on weight gain as single deficiency with an early reduction related to the absence of TLR2, and a later gain associated with a lack of TLR4.

Clinical features were monitored using a scoring matrix (Table S1) to determine the quantitative clinical effects of the absence TLR2 and 4 during infection (Figure 1B). Wt mice showed only minor clinical features of disease, developing a ruffled coat (from 5 dpi) and reduced weight gain (from 8 dpi). Infected TLR2^−/−^ mice developed clinical features more rapidly, with dull complexion and reduced weight gain observed as early as 3 dpi. Symptoms became more severe with reduced movement (from 5 dpi), hunched posture (from 6 dpi), and some tremors/laboured breathing (9–10 dpi). These features had only partially resolved by the end of the time course. By contrast, infected TLR4^−/−^ mice had a lack of clinical symptoms with ruffled fur at 11 dpi only. Clinical symptoms of TLR2/4^−/−^ mice were similar to those in TLR2^−/−^ pups until 7 dpi, after which they recovered.

Therefore, the absence of TLR2 leads to reduced weight gain and more severe clinical features of disease during *Chlamydia* respiratory infection in early life.

### Absence of TLR2 and 4 reduces the clearance of *Chlamydia* respiratory infection in early life

We then assessed whether the increases in disease in the absence of TLR2^−/−^ and 2/4^−/−^ correlated with *Chlamydia* numbers in the lung.

**Figure 2:**
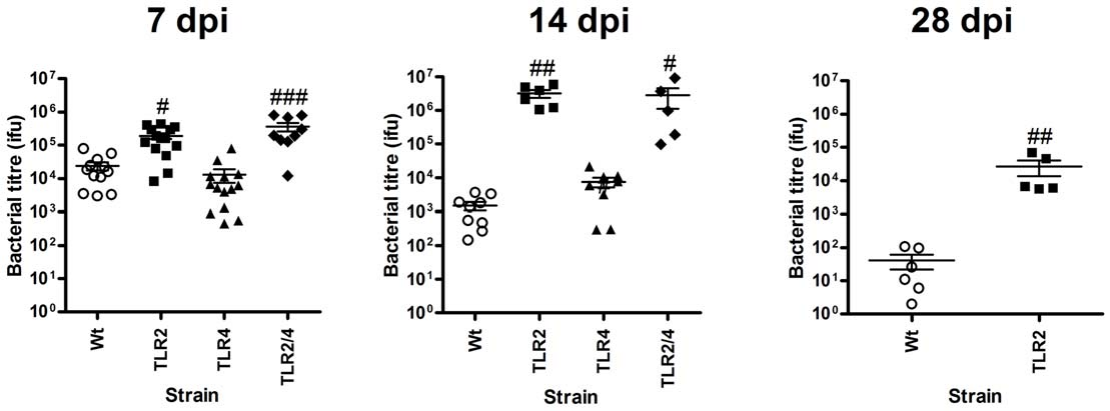
Absence of TLR2 reduces the clearance of *Chlamydia* respiratory infection in early life. Whole lungs were homogenized and DNA extracted. Quantitative real-time-PCR was used to determine the level of *Chlamydia* DNA in each sample relative to known standards. dpi = days post-inoculation, n = 5–9 mice/group. Results are presented as means ± SEM. # denotes significant difference between infected Wt and TLR^−/−^ groups. # p<0.05, ##p<0.01, ### p<0.001.

Infection, indicated by the number of *Chlamydia* inclusion-forming units (ifu) in the lung, in Wt mice was mild and peaked at 7 dpi, was substantially reduced (>1 log) by 14 dpi and almost cleared by 28 dpi (Figure 2). These results were similar to our previous observations [6]. The absence of TLR2 led to substantially (>2 fold) increased chlamydial *Chlamydia* numbers in the lung compared to infected Wt mice, which were still increasing at 14 dpi and remained high even at 28 dpi. The absence of TLR4 led to a small but significant increase in chlamydial *Chlamydia* numbers at 14 dpi. Infection in TLR2/4^−/−^ mice was similar to that in TLR2^−/−^ mice (Figure 2). These results demonstrate that TLR2 responses are predominantly responsible for the clearance of *Chlamydia* from the lung, and that TLR4 may contribute to complete resolution.

### Absence of TLR2 increases NK cell and neutrophil influx, and IL-17 expression in the lung during *Chlamydia* respiratory infection in early life

**Figure 3:**
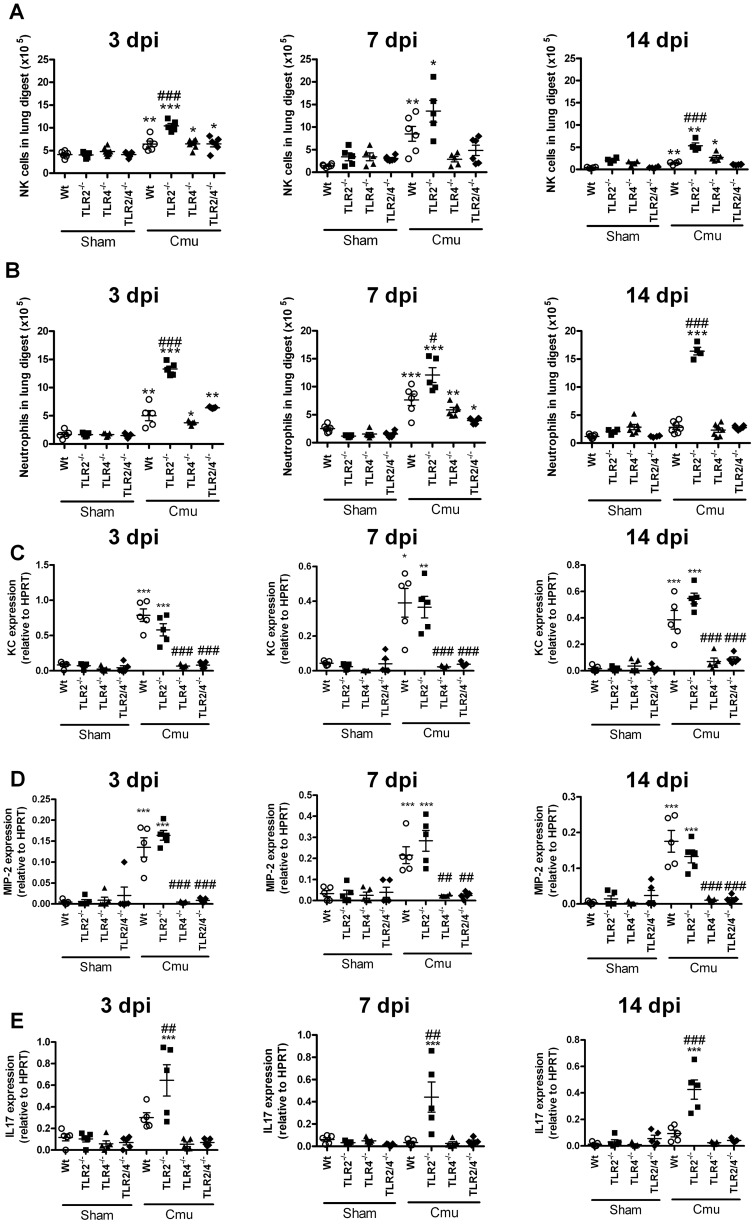
Absence of TLR2 increases NK and neutrophil influx, and IL-17 transcript levels in the lung during *Chlamydia* respiratory infection in early life. Flow cytometric analysis of single cell suspensions of lung tissue was used to determine the levels of NK cells and neutrophils in sham inoculated and infected Wt and TLR^−/−^ pups at 3, 7 and 14 dpi. (A) NK cells and (B) neutrophils were identified as cells expressing CD3^−^ and CD49^+^ and CD11b^+^ and GR-1^+^, respectively. Representative FACS plots are shown in Figure S2A and B. Total numbers of NK cells and neutrophils in the lung were calculated by multiplying total cell counts by the percentage of NK cells or neutrophils. Quantitative real-time-PCR was used to determine the transcript levels of (C) KC, (D) MIP-2 and (E) IL-17 in lung tissue at the same time points. dpi = days post-inoculation, n = 5–12 mice/group. Results are presented as means ± SEM. * denotes significant difference between sham inoculated and infected groups of the same strain at the same time point, # denotes significant difference between infected WT and TLR^−/−^ groups at the same time point. */#p<0.05, **p<0.01, ***/###p<0.001.

We then examined how the absence of TLR2 leads to more severe disease and infection. We first assessed the impact on innate inflammatory responses by measuring NK cells and neutrophils that protect against infection, over a time course. There were no differences in NK cell or neutrophil numbers in the lung between Wt and TLR^−/−^ sham inoculated control groups (Figures 3A and B and S2A and B).

Infection resulted in the recruitment of NK cells in all Wt and TLR^−/−^ mice, compared to sham inoculated controls, generally peaking at 3–7 dpi and then waning by 14 dpi (Figures 3A and S2A). The absence of TLR2, but not 4, resulted in significantly increased recruitment throughout the time course, compared to infected Wt mice.

Infection also resulted in the recruitment of neutrophils in all Wt and TLR^−/−^ mice, compared to sham inoculated controls, which then resolved in most strains (Figures 3B and S2B). The TLR2^−/−^ group was the exception as high levels of neutrophils were maintained throughout the time course compared to all other infected groups. Interestingly, neutrophil numbers increased to a small but significant degree in TLR4^−/−^ mice at 3 and 7 dpi, which was associated with effective control of infection (Figure 2).

To further investigate the differences in neutrophil infiltration in TLR2^−/−^ mice, we assessed the levels of mRNA transcript levels of keratinocyte-derived chemokine (KC) and macrophage-inflammatory protein-2 (MIP-2), (the mouse orthologs of human IL-8), and IL-17A which are the primary chemotactic signals for neutrophil infiltrates in *Chlamydia* and other respiratory infections in mice [9], [31]–[33]. *Chlamydia* infection resulted in increases in transcript levels of KC and MIP-2 to similar levels in the lungs of Wt and TLR2^−/−^, but not TLR4^−/−^ or 2/4^−/−^ mice, throughout the time course (Figure 3C and D). However, the increases in transcript levels in TLR2^−/−^ mice did not associate with increased influx of neutrophils in TLR2^−/−^ compared to Wt mice. By contrast, infection resulted in increased expression of IL-17A in TLR2^−/−^ mice, compared to all other infected groups throughout the time course, and was associated with the specific increase in neutrophil influx in this group (Figure 3E). We have previously shown that the transcript levels of these cytokines correlate with protein production in other respiratory infection models in mice [33].

Thus, the absence of TLR2 promotes enhanced innate responses (NK cell and neutrophil responses that may be driven by increased IL-17 expression), which are associated with increased severity of *Chlamydia* respiratory disease and infection in our early life infection model.

### Absence of TLR2 reduces early and increases later DC recruitment in the lung during *Chlamydia* respiratory infection in early life

**Figure 4:**
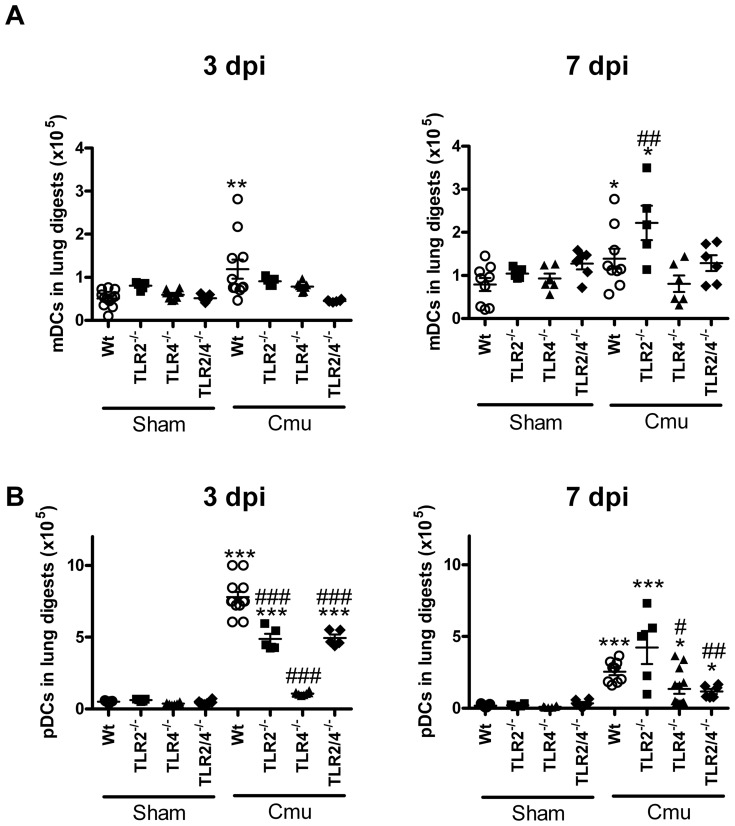
Absence of TLR2 increases late DC recruitment in the lung during *Chlamydia* respiratory infection in early life. Flow cytometric analysis of single cell suspensions of lung tissue was used to determine the levels of mDCs and pDCs in of sham inoculated and infected Wt and TLR^−/−^ pups at 3 and 7 dpi. (A) mDCs and (B) pDCs were identified as cells expressing CD11b^+^, CD11c^+^ and GR-1^−^, or CD11b^−^, B220^+^ and pDCA^+^, respectively. Representative FACS plots are shown in Figure S2C and D. dpi = days post-inoculation, n = 5–12 mice/group. Results are presented as means ± SEM. * denotes significant difference between sham inoculated and infected groups of the same strain at the same time point, # denotes significant difference between infected WT and TLR^−/−^ groups at the same time point. */#p<0.05, **/##p<0.01, ***/###p<0.001.

Since both innate and adaptive immunity contribute to the control of *Chlamydia* infection, we then investigated the impact of the absence of TLR2 on DCs that link innate and adaptive immune responses during the early stages of infection (3 and 7 dpi). There were no differences in mDC or pDC numbers in the lung between Wt and TLR^−/−^ sham inoculated groups (Figures 4A and B and S2C and D).

Infection of Wt mice induced low level early but sustained increases in mDCs, compared to sham inoculated controls (Figures 4A and S2C). TLR^−/−^ mice did not have increased mDC numbers early or late, with the exception of TLR2^−/−^ mice, which had later increases in mDCs compared to infected Wt and sham inoculated controls.

Infection of Wt mice induced pronounced early and sustained increases in pDCs, compared to sham inoculated controls (Figures 4B and S2D). TLR^−/−^ mice generally had increased pDCs numbers compared to sham inoculated groups but reduced numbers compared to infected Wt controls. However, TLR2^−/−^ mice had later increases in pDCs, although not statistically significantly so, compared to infected Wt controls.

Taken together these results show that the absence of TLR2 promotes reduced early and increased later DC responses that are associated with increased severity of *Chlamydia* respiratory disease and infection in early life.

### Absence of TLR2 increases the recruitment and activation of CD8^+^ T-cells in the lung during *Chlamydia* respiratory infection in early life

**Figure 5:**
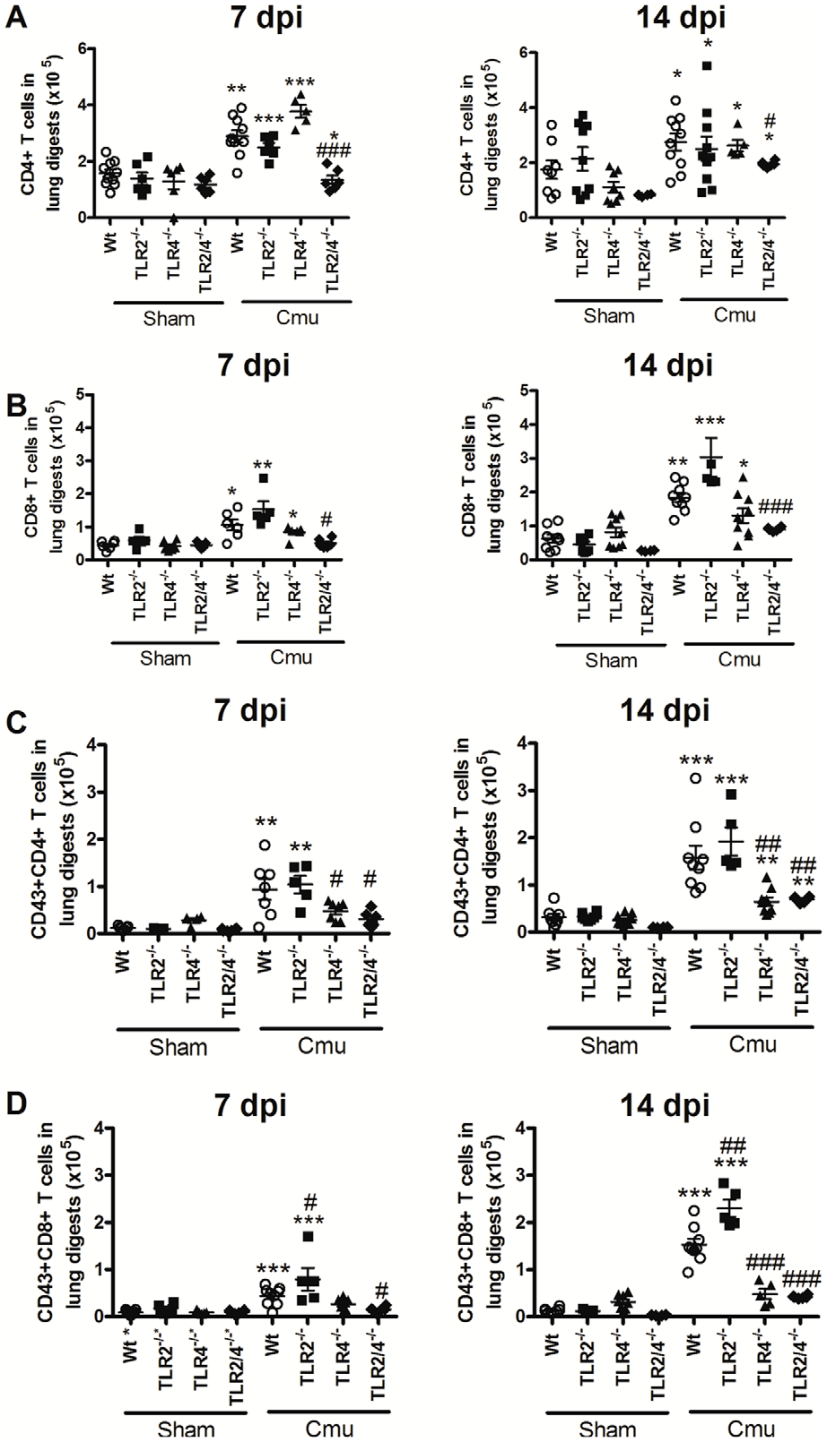
Absence of TLR2 increases activated CD8^+^ T-cell recruitment in the lung during *Chlamydia* respiratory infection in early life. Flow cytometric analysis of single cell suspensions of lung tissue was used to determine the levels of total and activated CD4^+^ or CD8^+^ T-cells in sham inoculated and infected Wt and TLR^−/−^ pups at 7 and 14 dpi. T-cell populations were identified by selecting those cells, which were CD3^+^ and CD4^+^ or CD8^+^. These populations of (A) total CD4^+^ and (B) total CD8^+^ T-cells were then analysed for CD43 expression, as a marker of T-cell activation (C and D). Representative FACS scatter plots of these data are shown in Figure S2E and F. dpi = days post-inoculation, n = 6–12 mice/group. Results are presented as means ± SEM. * denotes significant difference between sham inoculated and infected groups of the same strain at the same time point, # denotes significant difference between infected WT and TLR^−/−^ groups at the same time point. #p<0.05, **/##p<0.01, ***/###p<0.001.

Since T-cells have important roles in the control of *Chlamydia*, we then investigated the impact of the absence of TLR2 on adaptive CD4^+^ and CD8^+^ T-cell infiltration and activation during the later stages of infection (7 and 14 dpi). There were no differences in CD4^+^ and CD8^+^ T-cell numbers or activation (CD43 expression) in the lung between Wt and TLR^−/−^ sham inoculated groups (Figures 5A and B and S2E and F).

Infection induced significant increases in CD4^+^ and activated CD4^+^ T-cells in the lungs of Wt and all TLR^−/−^ groups (Figures 5A and C and S2E), compared to sham inoculated controls. The only differences between groups were that the absence of TLR4 led to reduced recruitment (TLR2/4^−/−^ group) and/or activation (TLR4^−/−^ and TLR2/4^−/−^ groups) of CD4^+^ cells compared to infected Wt groups.

Infection induced significant increases in CD8^+^ and activated CD8^+^ T-cells in the lungs of infected Wt and TLR^−/−^ groups (Figures 5B and D and S2F). The levels of activated CD8^+^ T-cells were greater in TLR2^−/−^ groups and reduced in the absence of TLR4^−/−^ compared to infected Wt mice.

Thus, the absence of TLR2 increases the recruitment of activated of CD8^+^ T-cells in the lung, which is associated with increased severity of *Chlamydia* respiratory disease and infection in early life.

### Absence of TLR2 and 4 reduces IFNγ release and proliferation of *ex-*vivo stimulated T-cells during *Chlamydia* respiratory infection in early life

The release of IFNγ and the proliferation of T-cells that release this and other cytokines are important factors in the clearance of *Chlamydia* [6], [11], [23], [25], [26], therefore, we then investigated the impact of the absence of TLR2 and 4 on these responses during the later stages of disease (14 dpi). There were no differences in these factors between un-infected Wt and TLR^−/−^ sham inoculated groups (Figur 6).

**Figure 6:**
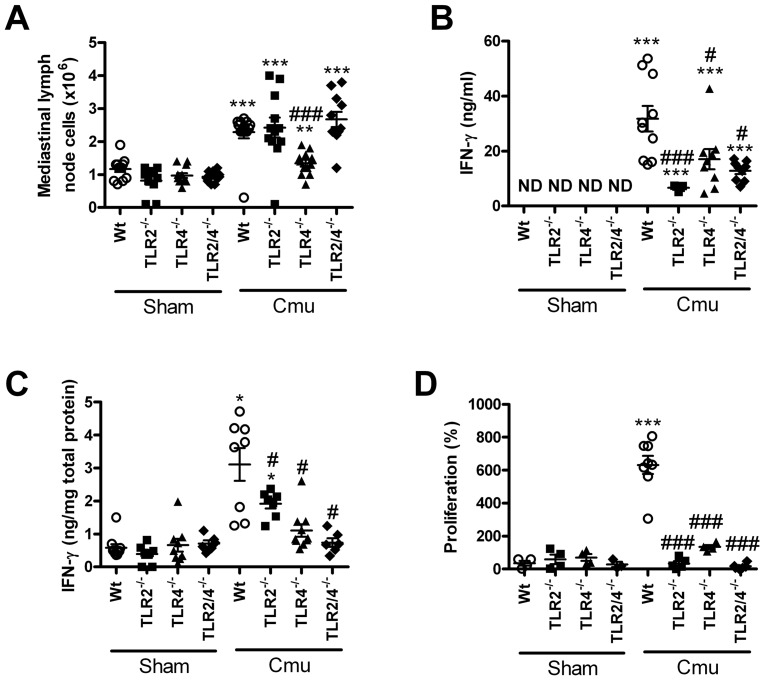
Absence of TLR2 and 4 reduces IFNγ release and proliferation of *ex-*vivo stimulated T-cells during *Chlamydia* respiratory infection in early life. Single cell suspensions of mediastinal lymph nodes were prepared from sham inoculated and infected Wt and TLR^−/−^ pups at 14 dpi. (A) Total cell numbers in lung-draining mediatinal lymph nodes were determined by trypan blue exclusion. (B) Other mediatinal lymph node cells were stimulated with media only or MOMP and IFNγ levels in culture supernatants were determined by ELISA. (C) IFNγ protein levels in lung homogenates were also quantitated by ELISA. (D) Lymph node cell proliferation was assessed following stimulation with MOMP (2 µg/ml). Percentage proliferation was calculated relative to cells stimulated with media only. n = 6–12 mice/group. Results are presented as means ± SEM. * denotes significant difference between sham inoculated and infected groups of the same strain, # denotes significant difference between infected WT and TLR^−/−^ groups. */#p<0.05, **p<0.01, ***/###p<0.001.

The number of cells recovered from mediastinal lymph nodes was approximately double in infected Wt, TLR2^−/−^ and 2/4^−/−^ mice compared to sham inoculated controls (Figure 6A). By contrast there was only a small increase in TLR4^−/−^ mice.

When stimulated with the major outer membrane protein (MOMP), the immunodominant antigen of *C. muridarum*, mediastinal lymph node cells from infected Wt mice released a significant amount of IFNγ, compared to media only (Figure 6B). Cells from infected TLR^−/−^ groups also released IFNγ but to a reduced level compared to Wt controls.

Infected Wt mice had increased levels of IFNγ protein in the lung compared to sham inoculated mice (Figure 6C). Infected TLR2^−/−^ mice also had increased IFNγ protein, but this was significantly reduced compared to infected Wt controls. TLR4^−/−^ and TLR2/4^−/−^ mice had no increase in IFNγ levels.

When stimulated with MOMP, mediastinal lymph node cells from infected Wt mice proliferated profusely, compared to sham inoculated controls (Figure 6D). By contrast cells from TLR^−/−^ groups did not proliferate.

Thus, the absence of TLR2 and 4 reduces IFNγ release in the lymph nodes and lungs and lymph node T-cell proliferation that correlate with increased severity of *Chlamydia* respiratory disease and infection in early life.

### Absence of TLR2 reduces phagocytosis of *Chlamydia* by neutrophils

**Figure 7:**
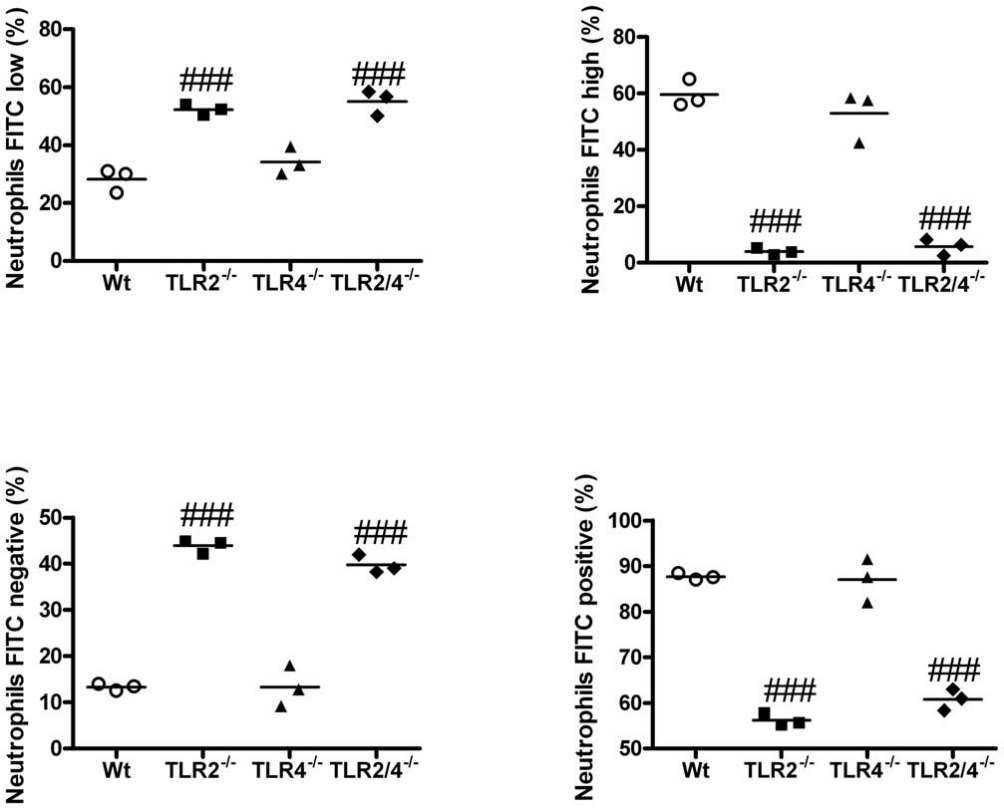
Absence of TLR2 reduces phagocytosis of *Chlamydia* by neutrophils. Bone marrow was collected from Wild type (Wt), and TLR^−/−^ mice, grown, and non- and semi-adherent cells cultured with fluorescently (FITC) labelled latex beads. Neutrophils were identified as expressing CD11b^+^ and GR1^+^. The percentage of neutrophils that were FITC low, high, negative or positivewere determined. FACS scatter plots of these data are shown in Figure S3. n = 3 replicates, results are presented as means ± SEM. * denotes significant different between WT and TLR^−/−^ groups. ***p<0.001.

Neutrophils play a critical role in the clearance of *Chlamydia* [34]. However, we show that in the absence of TLR2 both infection and neutrophil levels are increased. This suggests that TLR2 on neutrophils is important for the effective clearance of *Chlamydia*. To further investigate the role of TLR2 in neutrophil clearance Wt and TLR^−/−^ neutrophils were cultured from bone marrow and incubated with fluorescently labelled latex beads. The phagocytic capacity of neutrophils to take up latex beads was assessed using flow cytometry. Both Wt and TLR4^−/−^ neutrophils were able to effectively phagocytose latex beads (Figures 7 and S3). In stark contrast TLR2^−/−^ and 2/4^−/−^ neutrophils had a markedly reduced phagocytic capacity.

Thus, the absence of TLR2 suppresses phagocytosis by neutrophils and provides a mechanism whereby the absence of TLR2 leads to increased severity of *Chlamydia* respiratory disease, infection and inflammatory responses in early life.

## Discussion

Understanding the mechanisms associated with immune responses to *Chlamydia* respiratory infection in early life is important in developing targets for effective vaccination and therapeutic strategies for infection. Identification of the pivotal components of inflammation induced by *Chlamydia* will also facilitate the elucidation of its role in the pathogenesis of chronic childhood diseases, particularly asthma, as well as diseases of later life such as atherosclerosis, which may have their origins in early life.

Here we demonstrate that TLR2 is critical for defence against *Chlamydia* respiratory tract infection in early life (Figures 1, 2, 3, 4, 5, 6, 7 and S1, S2, S3). Wt mice had mild disease and infection, mounting a sufficient early innate response and a subsequent influx of activated CD4^+^ and CD8^+^ T-cells, effective release of *Chlamydia*-specific IFNγ and proliferation of mediastinal lymph node T-cells. Together these responses controlled the infection. By contrast, TLR2^−/−^ mice suffered more severe disease (reduced weight gain, clinical features), infection and more intense and prolonged inflammation (enhanced NK cell, neutrophil, DC and activated CD8^+^ T-cell influx and IL-17 responses). They also had reduced protective IFNγ and adaptive (cell responses in lymph nodes) immune responses. TLR4^−/−^ mice did not suffer overt disease or substantial induction of inflammatory responses, however, infection was elevated at 14 dpi suggesting that TLR4 is important in later clearance of *Chlamydia*. TLR2/4^−/−^ mice had features of single TLR deficiencies with severe early disease but were able to resolve symptoms, even though infection persisted. In these double deficient mice resolution of disease and inflammatory responses (but not infection) was achieved with reduced innate and adaptive responses. Critically the absence of TLR2 in neutrophils abrogated their phagocytic capacity, which provides a mechanism for loss of control of infection and subsequent enhanced inflammatory responses. The lack of resolution of disease and inflammation in TLR2^−/−^, but not 2/4^−/−^, mice suggests that the enhanced but ineffective inflammatory responses were TLR4-mediated. These enhanced responses failed to diminish bacterial load, and increased and prolonged disease symptoms by inducing inflammatory damage.

TLR2 and 4 are commonly regarded as the primary pattern recognition receptors responsible for the detection of Gram positive and negative bacteria, respectively. However, we found that the immune response to *Chlamydia* in early life involved contributions of both TLR2 and 4, as well as potentially other PRRs. Our previous studies have shown that the peak of *Chlamydia* infection in early life occurs around 10–15 dpi with reduced weight gain occurring as early 7 dpi [6]. The current study shows that TLR2 protects against the early stages of infection by inducing innate immune responses (e.g. NK cells and neutrophil influx), but if infection is not controlled, as in TLR2^−/−^ mice, TLR4 mediates subsequent inflammatory and adaptive responses (involving DCs and activated CD8+T-cells) (Figures 1, 2, 3, 4, 5 and S2). Together uncontrolled infection and enhanced but ineffective inflammation induces increased severity and persistence of infection and disease that is most prominent in the absence of TLR2 combined with the presence of TLR4 i.e. in TLR2^−/−^ compared to Wt and TLR4^−/−^ or 2/4^−/−^ mice.

Infected Wt mice show two significant periods of infection-induced disease compared to sham inoculated controls (Figures 1 and 2). The first commences at 4 dpi and involves increased clinical symptoms and infection, and is likely to be an effect of tissue damage resulting from the peak of the innate response. The second, from 11–14 dpi, involves reduced weight gain and correlates with maximal adaptive immune responses and inflammation. TLR2^−/−^ mice show a more immediate and dramatic induction of disease symptoms, which persisted throughout the time course, and are likely to result from the early and late influx of innate inflammatory and adaptive immune cells, respectively. In stark contrast, TLR4^−/−^ mice remained largely asymptomatic up to 14 dpi, which probably resulted from the clearance of infection through reduced but adequate innate and adaptive responses. TLR2/4^−/−^ mice were of an intermediate disease phenotype.

NK cells and neutrophils are amongst the first immune cells to arrive at a site of bacterial infection, migrating from the blood in high numbers. Neutrophils express TLR1, 2, 4, 5 and 6 and the stimulation of each promotes activation [35]. Activation of TLRs can recruit and activate neutrophils directly or indirectly *via* the up-regulation of adhesion molecules or cytokines released from macrophages, endothelial or epithelial cells. Thus, TLR deficiency on other cells may also contribute to the changes in neutrophil responses in our studies. Nevertheless, our results show that a deficiency in TLR2 resulted in increased and persistent (to 14 dpi) neutrophil influx into the lung (Figure 3). Prolonged neutrophil presence is accompanied by increases in the pro-inflammatory mediators they secrete, which are potent contributors to inflammatory damage and disease outcomes. Our observations contrast with those of Rodriguez *et* *al*., who found that neutrophil recruitment in response to *C. pneumoniae* infection in adult mice, was dependent on TLR2 and not 4 [18]. Naiki *et* *al*., however, reported that neither TLR2 nor 4 affected the magnitude or kinetics of neutrophil recruitment during adult *C. pneumoniae* infection [17]. These authors did, however, show a delayed and reduced neutrophil recruitment in the absence of MyD88, the major adaptor protein in the TLR2 and 4 signalling pathways. This led to the hypothesis that a redundancy exists between TLR2 and 4 MyD88-dependent pathways, with one able to compensate in the absence of the other. It is possible that our findings do not correlate with these previous studies since we focused on neonatal infection. Thus, the compensatory MyD88-dependent pathways may be underdeveloped in neonatal mice and the ability of TLR2 and 4 to compensate may only be developed in later life. Furthermore, *C. pneumoniae*, used in previous studies, is not a natural mouse pathogen, and may not induce a productive infection or represent a natural host-pathogen relationship.

DCs are the primary antigen presenting cells responsible for communication between innate and adaptive immune responses. Our study shows that they are involved in the innate response to *C. muridarum* infection in early life (Figure 4). mDCs, expressing TLR1, 2, 4 and 6 have been primarily implicated in immunity against extracellular bacteria, whereas pDCs, that express mainly TLR7 and 9, are regarded as important in protection against viruses and intracellular pathogens. This study provides evidence contrary to the classical paradigm, since both mDC and pDC numbers were increased in the lungs of *C. muridarum* infected Wt mice at 3 and 7 dpi, with pDCs predominating. This may be due to the unique structure of the intracellular atypical *Chlamydia*, or different responses that occur in early life. Notably, the absence of TLR2 resulted in a later increase in DCs (7 dpi). These DCs may further enhance adaptive immune responses, inflammation and inflammatory damage in TLR2^−/−^ mice. Our observations contrast with the *in vitro* results obtained by Prebeck *et* *al*., who used bone marrow-derived DCs from TLR2^−/−^ and 4^−/−^ mice to demonstrate that TLR2 was more important than TLR4 in the activation of DCs in response to *C. pneumoniae* [36]. Again the use of *C. pneumoniae* as opposed to *C. muridarum* may impact on the outcome, but it is also likely that in a whole organism, signalling that results from TLR expression on other cells may influence DC activation and recruitment. pDCs do not express TLR2 or 4, however, our results suggest that the absence of TLR2 influences pDC recruitment, potentially through the loss of control of infection that enhances inflammatory responses.

Both CD4^+^ and CD8^+^ T-cells contribute to the clearance of *Chlamydia* in adult respiratory infection [23], [25]. We show that CD4^+^ and CD8^+^ T-cells are recruited and activated in the lung during neonatal infection (Figure 5). There were no increases in activated CD4^+^ and CD8^+^ T-cells in TLR2/4^−/−^ or TLR4^−/−^ mice, even though TLR2/4^−/−^ mice had a high level of persistent infection. This demonstrates that TLR4 signalling is vital for the activation of T-cells during infection. Activated CD8^+^ T-cells were further increased in the absence of TLR2^−/−^, which correlated with enhanced inflammation and DC responses.

Cytokine profiles of T-cells are crucial in determining their role in immune responses [12], [31]. IL-17 is released by a range of innate and adaptive immune cells, it is important in driving neutrophilic inflammation and is induced by *Chlamydia* respiratory infection [9], [31]–[33]. IL-17A transcript levels were significantly increased in the lungs of TLR2^−/−^ mice, in response to enhanced infection and inflammation and may contribute to increased and persistent neutrophilic inflammation in these mice (Figure 3E). IFNγ production is essential for the clearance of *Chlamydia* [6], [11], [23], [25], [26]. Levels of IFNγ secreted in lymph node cultures stimulated with MOMP is representative of the number of IFNγ producing *C. muridarum* specific T-cells, and were increased in infected Wt mice (Figure 6B). IFNγ levels were significantly reduced in all infected TLR^−/−^ strains, most significantly in TLR2^−/−^ mice, and may have contributed to enhanced infection in TLR2^−/−^ pups. These responses were antigen-specific as there were no differences in non-specific stimulation with anti-CD3/CD28 (data not shown). Levels of lymph node hyperplasia did not correlate with levels of secreted IFNγ in mice TLR2^−/−^ mice (Figure 6A). This may be due to inappropriate T cell activation or induction of Th2 cells in lymph nodes. This would lead to increases in the number of cells in the lymph nodes but with reduced release of IFNγ. The levels of IFNγ in lung homogenates are representative of the phenotype of the T-cell response in the lungs. Infected Wt mice had increased IFNγ expression in lung homogenates (14 dpi) (Figure 6C). TLR2 deficiency significantly reduced IFNγ expression even though the numbers of total and activated T-cells were similar, again suggesting inappropriate T cell activation or decrease in IFNγ -producing T cells. However, the persisting innate immune cells in the lungs of TLR2^−/−^ mice may also contribute to IFNγ secretion. TLR4 and 2/4 deficiency resulted in IFNγ levels no greater than those in un-infected controls, reflecting the lack of inflammation. Re and colleagues [37] have previously shown *in vitro* that stimulation of TLR4 on DCs, leads to the secretion of IFNγ. Thus, the decrease in inflammation in the absence of TLR4 is possibly due to reduced levels of the proinflammatory cytokine IFNγ. Collectively these results suggest that TLR2 is required to induce a protective Th1-mediated adaptive immune response in the lung in response to neonatal *Chlamydia* infection.

We have previously shown that *C. muridarum* employs similar mechanisms of pathogenicity to *C. pneumoniae* in humans, and that the time-course, immunological and histopathological progression of disease closely resembles that observed in human *C. pneumoniae* infection [6], [8]. *Chlamydia* can infect innate immune cells including neutrophils and DCs [34], which may alter their function and enable *Chlamydia* to persist, and induce TLR4-mediated inflammation. Prolonged infection may lead to the formation of persistent bodies, resulting in asymptomatic persistent infection, which can persist for many years and cause continued damage and prolonged neutrophil influx over time. Indeed chronic, persistent infections with *C. pneumoniae* in the lung are implicated in the pathogenesis of asthma and atherosclerosis [1], [38]. The persistent neutrophil influx into the lungs of TLR2^−/−^ mice may result from the inability of neutrophils to undergo apoptosis, however, we show that TLR2^−/−^ neutrophils have reduced phagocytic capacity (Figure 7), and this may be the mechanism that leads to persistent infection (possibly including within the compromised neutrophils) and excessive and prolonged neutrophil recruitment. TLR2 may also be important in enhancing oxidative bactericidal activity of neutrophils. Indeed, TLR2 is important in the phagocytosis and killing of *Streptococcus pneumoniae* by neutrophils through the enhancement of oxidative bactericidal activity [39]. TLR2 is also important in macrophage phagocytosis of some bacteria (e.g. *Listeria monocytogenes* [40]) but not others (e.g. *Borellia burgdorferi* [41]). Our results suggest that TLR2 expression on macrophages is less important than on neutrophils in *Chlamydia* respiratory infections in early life. TLR2 is also important in the phagocytosis of fungi (*Aspergillus* fumigatus) [42], however, other studies show that this TLR may promote yeast infections, which results from increases in regulatory T cells [43] that have potent anti-inflammatory properties [44]–[46]. This suggests that in some situations involving infections, TLR2 may have anti-inflammatory effects.

Vaccines are being developed to protect against *Chlamydia* infection [47]–[48], and recently TLR2 agonists have been used as effective adjuvants for anti-*Chlamydia* vaccines in mice [49]. Collectively, our results and other studies suggest that TLR2 agonists may have beneficial roles in enhancing host immunity and the clearance of infection. They may be effective therapeutics against infectious agents and be an alternative treatment for antibiotic resistant bacterial infections.

In summary, in *Chlamydia* respiratory infection in early life, it is likely that TLR2 responses are induced very early and control infection. A lack of control in the absence of TLR2 results in enhanced infection that drives greater TLR4-mediated inflammatory responses, including maintenance of high levels of NK cells, neutrophils, DCs and T-cells. TLR2 deficiency leads to reduced phagocytic activity of neutrophils and a loss of control of infection and inflammation. TLR2 agonists may have beneficial effects in the treatment of *Chlamydia* and other microbial infections.

## Materials and Methods

### Ethics statement

This study was performed in strict accordance with the recommendations in the Australian code of practice for the care and use of animals for scientific purposes issued by the National Health and Medical Research Council of Australia. All protocols were approved by the Animal Ethics Committee of The University of Newcastle and all efforts were made to minimise suffering.

### Mice

Female, pregnant Wt, TLR2^−/−^, 4^−/−^, and 2/4^−/−^ BALB/c mice were obtained from the Animal Services Unit, The University of Newcastle or the John Curtin School of Medical Research, at 14 days gestation. To maximise the likelihood of simultaneous litters, female mice were estrus synchronized by exposure to male pheromones for 3 days prior to mating. Pregnant mice were monitored twice daily to determine the date of partruition. All animals were maintained under specific pathogen-free conditions.

### Infection, weight monitoring, clinical score, and sample collection

Two days after birth, pups were inoculated intra-nasally with 400 ifu of *C. muridarum* in sucrose phosphate glutamate (SPG, 5 µl) buffer [6], [8]. Controls were sham inoculated with SPG. Pups were weighed prior to inoculation and weight and clinical features were monitored daily thereafter. Mice were weighed individually, while clinical score was assessed based on presentation of whole litters. Assessment of clinical symptoms of infection was based on weight, appearance and activity level (Table S1). Mice were euthanized 3, 7,14 and 28 dpi (i.e. 5, 9, 16 and 30 days of age) by decapitation under isofluorane anaesthesia (age <16 days) or sodium pentobarbitone overdose (at age 16 days).

### Chlamydia recovery

Whole lungs were removed and stored at −80°C. DNA extractions were performed and *C. muridarum* numbers (ifu) determined in whole lungs by real-time quantitative PCR and by comparison with known standards as we have previously described [6], [8]–[10].

### Inflammatory cells in the lung

Single cell suspension of lungs were prepared [44], and incubated (1x10^6^ cells/well in 96 well plates, 20 min, 4°C) with Fc block (10 ng/ml, BD Biosciences, North Ryde, NSW, Australia) in FACS buffer (2% fetal calf serum in PBS) [8]. Cell surface antigens were labelled by incubation (30 min, 4°C) with fluorochrome (FITC, PE, PerCP, PerCpCy5.5 or APC) conjugated antibodies (Tables S2 and S3). Cells were analysed using a FACS Canto flow cytometer and FACSDiva software (BD Biosciences). Cell populations were selected based on forward scatter (FSC) and side scatter (SSC) and were defined as; NK cells (low, moderate/high FSC, low/moderate SSC, CD3^−^CD49b^+^), neutrophils (high FSC, moderate SSC, CD11b^+^GR-1^hi^), mDCs (moderate/high SSC, low/moderate FSC, CD11b^+^CD11c^+^), pDCs (moderate FSC, low/moderate SSC, CD45R/B220^+^CD11b^−^PDCA^+^), CD4 T cells (low/moderate/high FSC, low SSC, CD3^+^CD4^+^) or CD8 T-cells (low/moderate/high FSC, low SSC, CD3^+^CD8^+^) [9], [32], [50]–[53]. CD43 was used as a marker of activation of T-cell populations [54].

### KC, MIP-2 and IL-17 expression in lung homogenates

Lungs were excised and stored in RNA-later (Ambion, TX, USA) at −80°C. Tissues were homogenized, extracted and KC, MIP-2 and IL-17 transcript levels determined by quantitative real-time PCR as we have previously described [9], [32].

### Lymph node hyperplasia

Single cell suspensions of mediastinal lymph nodes were prepared as we have described [55], by gently pressing lymph nodes through a 70 μm nylon cell strainer and washing with excess ice-cold Hanks buffered salt solution (HBSS; Trace Scientific, Noble Park, NSW). Contaminating erythrocytes were removed by incubation with red blood cell lysis buffer. After washing, total cell numbers were determined using trypan blue exclusion with a haemocytometer (Neubauer, Dusseldorf, Germany) and light microscope.

### Lymph node T-cell stimulation and IFNγ release

Mediastinal lymph node T-cells (5x10^5^) were stimulated with media or MOMP (20 μg/ml), and IFNγ release into culture supernatants determined by ELISA as we have previously described [6], [8], [9], [48], [56].

### IFNγ protein in the lung

Whole lungs were homogenized in tissue lysis buffer (500 μl, 0.5% Triton-X, 150 mM NaCl, 15 mM Tris, 1 mM CaCl_2_, 1 mM MgCl2, in PBS) using a Tissue Tearor (BioSpec Products, Oklahoma, USA), incubated on ice for 30 min and centrifuged at 10,000 *g* for 20 min [33]. IFNγ proteins concentrations were determined by ELISA.

### T-cell proliferation

Mediastinal lymph node cells (5x10^5^) were plated and stimulated with media or MOMP (20 μg/ml), and incubated for 72 h. Cell proliferation was then assessed using CellTiter 96® Non-Radioactive Cell Proliferation assay kits (MTT, Promega, Maddison, WI, USA), following the manufacturer's instructions. Cell proliferation was calculated as the percentage increase in proliferation in stimulated relative to unstimulated samples as we have previously described [56].

### Phagocytosis

Bone marrow was collected from Wt and TLR^−/−^ mice [10], cultured, and non- and semi-adherent cells incubated with fluorescently (FITC) labelled latex beads for 24 h, as per manufacturer's instructions for flow cytometry (Phagocytosis assay kit, IgG FITC, Cayman Chemicals, Ann Arbour, MI, USA). Neutrophils were then identified as expressing CD11b^+^ and GR1^+^. The percentage of neutrophils that were FITC low, high, negative or positive were determined.

### Statistics

Differences between repeated measurements (weight and clinical score) were analysed using one-way repeated measures ANOVA with Dunnet's post-test. All other differences between groups were analysed using a one-way ANOVA with Tukey's multiple comparison post-test using Graphpad Prism version 4.0 software (Graphpad, La Jolla, CA, USA). P values <0.05 were considered significant. Data points are presented as means ± SEM from 6–12 replicates, unless otherwise specified.

## Supporting Information

Figure S1 Absence of TLR2 does not affect growth in sham inoculated mice, but infection of Wt mice reduces weight gain. (A) Wild type (Wt) and TLR^−/−^ pups were sham inoculated and weighed individually each day. The percentage weight gain was calculated daily relative to the average weight of the litter prior to inoculation. (B) Wt mice were sham inoculated or infected and weight gain monitored. n = 10–12 pups/group (5–6 pups/litter). Results are presented as means ± SEM. * denotes significant difference between sham inoculated and infected Wt groups. *** p<0.001 for the whole curves.
(TIF)

Figure S2 Absence of TLR2 increases NK and neutrophil influx and later DC and activated CD8+ T-cell recruitment during *Chlamydia* respiratory infection in early life. Representative FACS scatter plots of flow cytometric analysis of samples (A) NK Cells (B) Neutrophils (C) mDCs (D) pDCs (E) Activated CD4^+^ T-cells and (F) Activated CD8^+^ T-cells, as described in Figures 3–5, at 7 dpi.
(TIF)

Figure S3 Absence of TLR2 reduces phagocytosis of *Chlamydia* by neutrophils. Representative FACS scatter plots of flow cytometric analysis of samples described in Figure 7. The numbers of low and high FITC-containing neutrophils were combined to produce the “Neutrophils FITC positive (%)” panel in Figure 7.
(TIF)

Table S1 Criteria used to assess clinical scores.
(TIF)

Table S2 Fluorescently-labeled cell surface markers used to identify cell populations.
(TIF)

Table S3 Fluorescently-labeled antibodies used in flow cytometry.
(TIF)

## References

[1] Hansbro P, Beagley K, Horvat J, Gibson P (2004). Role of atypical bacterial infection of the lung in predisposition/protection of asthma.. Pharmacol Ther.

[2] Principi N, Esposito S, Blasi F, Allegra L, group Ms (2001). Role of Mycoplasma pneumoniae and Chlamydia pneumoniae in children with community-acquired lower respiratory tract infections.. Clin Infect Dis.

[3] Webley W, Salva P, Andrzejewski C, Cirino F, West C (2005). The bronchial lavage of pediatric patients with asthma contains infectious Chlamydia.. Am J Respir Crit Care Med.

[4] Webley W, Tilahun Y, Lay K, Patel K, Stuart E (2009). Occurrence of *Chlamydia trachomatis* and *Chlamydia pneumoniae* in paediatric respiratory infections.. Eur Respir J.

[5] Blasi F, Cosentini R, Tarsia P (2000). *Chlamydia pneumoniae* respiratory infections.. Curr Opin Infect Dis.

[6] Horvat J, Beagley K, Wade M, Preston J, Hansbro N (2007). Neonatal chlamydial infection induces mixed T-cell responses that drive allergic airway disease.. Am J Respir Crit Care Med.

[7] Kaiko G, Phipps S, Hickey D, Lam C, Hansbro P (2008). Chlamydia muridarum infection subverts dendritic cell function to promote Th2 immunity and airways hyperreactivity.. J Immunol.

[8] Horvat J, Starkey M, Kim R, Phipps S, Gibson P (2010). Early-life chlamydial lung infection enhances allergic airways disease through age-dependent differences in immunopathology.. J Allergy Clin Immunol.

[9] Horvat J, Starkey M, Kim R, Beagley K, Preston J (2010). Chlamydial respiratory infection during allergen sensitization drives neutrophilic allergic airways disease.. J Immunol.

[10] Asquith K, Horvat J, Kaiko G, Carey A, Beagley K (2011). Interleukin-13 promotes susceptibility to chlamydial infection of the respiratory and genital tracts.. PLoS Pathog.

[11] Jupelli M, Murthy A, Chaganty B, Guentzel M, Selby D (2011). Neonatal chlamydial pneumonia induces altered respiratory structure and function lasting into adult life.. Lab Invest.

[12] Kaiko G, Horvat J, Beagley K, Hansbro P (2008). Immunological decision-making: how does the immune system decide to mount a helper T-cell response?. Immunology.

[13] Schwandner R, Dziarski R, Wesche H, Rothe M, Kirschning C (1999). Peptidoglycan- and lipoteichoic acid-induced cell activation is mediated by toll-like receptor 2.. J Biol Chem.

[14] Akira S, Takeda K (2004). Toll-like receptor signalling.. Nat Rev Immunol.

[15] Derbigny W, Kerr M, Johnston R (2005). Pattern recognition molecules activated by Chlamydia muridarum infection of cloned murine oviduct epithelial cell lines.. J Immunol.

[16] Sasu S, LaVerda D, Qureshi N, Golenbock D, Beasley D (2001). Chlamydia pneumoniae and chlamydial heat shock protein 60 stimulate proliferation of human vascular smooth muscle cells via toll-like receptor 4 and p44/p42 mitogen-activated protein kinase activation.. Circ Res.

[17] Naiki Y, Sorrentino R, Wong M, Michelsen K, Shimada K (2008). TLR/MyD88 and liver X receptor alpha signaling pathways reciprocally control Chlamydia pneumoniae-induced acceleration of atherosclerosis.. J Immunol.

[18] Rodriguez N, Wantia N, Fend F, Dürr S, Wagner H (2006). Differential involvement of TLR2 and TLR4 in host survival during pulmonary infection with *Chlamydia pneumoniae*.. Eur J Immunol.

[19] He X, Nair A, Mekasha S, Alroy J, O'Connell C (2011). Enhanced virulence of Chlamydia muridarum respiratory infections in the absence of TLR2 activation.. PLoS One.

[20] Zhang P, Summer W, Bagby G, Nelson S (2000). Innate immunity and the pulmonary host defense.. Immunol Rev.

[21] Lambrecht B, Prins J, Hoogsteden H (2001). Lung dendritic cells and host immunity to infection.. Eur Respir J.

[22] Steinman R (1991). The dendritic cell system and its role in immunogenicity.. Annu Rev Immunol.

[23] Rottenberg M, Gigliotti Rothfuchs A, Gigliotti D, Svanholm C, Bandholtz L (1999). Role of innate and adaptive immunity in the outcome of primary infection with Chlamydia pneumoniae, as analyzed in genetically modified mice.. Journal of Immunology.

[24] Williams D, Grubbs B, Pack E, Kelly K, Rank R (1997). Humoral and cellular immunity in secondary infection due to murine Chlamydia trachomatis.. Infect Immun.

[25] Rothfuch A, Kreuger M, Wigzell H, Rottenberg M (2004). Macrophages, CD4+ or CD8+ cells are each sufficient for protection against Chlamydia pneumoniae infection through their ability to secrete IFN-gamma.. J Immunol.

[26] Jupelli M, Guentzel M, Meier P, Zhong G, Murthy A (2008). Endogenous IFN-gamma production is induced and required for protective immunity against pulmonary chlamydial infection in neonatal mice.. J Immunol.

[27] Hansbro N, Horvat J, Wark P, Hansbro P (2008). Understanding the mechanisms of viral induced asthma: new therapeutic directions.. Pharmacol Ther.

[28] Culley F, Pollott J, Openshaw P (2002). Age at first viral infection determines the pattern of T cell-mediated disease during reinfection in adulthood.. J Exp Med.

[29] Adkins B, Bu Y, Guevara P (2001). The generation of Th memory in neonates versus adults: prolonged primary Th2 effector function and impaired development of Th1 memory effector function in murine neonates.. J Immunol.

[30] Kovarik J, Siegrist C (1998). Immunity in early life.. Immunol Today.

[31] Hansbro P, Kaiko G, Foster P (2011). Cytokine/anti-cytokine therapy – novel treatments for asthma?. Br J Pharmacol.

[32] Essilfie A, Simpson J, Dunkley M, Morgan L, Oliver B (2012). Combined *Haemophilus influenzae* respiratory infection and allergic airways disease drives chronic infection and features of neutrophilic asthma.. Thorax In press.

[33] Essilfie A, Simpson J, Horvat J, Preston J, Dunkley M (2011). Haemophilus influenzae infection drives IL-17-mediated neutrophilic allergic airways disease.. PLoS Pathog.

[34] Beagley K, Huston W, Hansbro P, Timms P (2009). Chlamydial infection of immune cells: altered function and implications for disease.. Crit Rev Immunol.

[35] Hayashi F, Means T, Luster A (2003). Toll-like receptors stimulate human neutrophil function.. Blood.

[36] Prebeck S, Kirschning C, Dürr S, da Costa C, Donath B (2001). Predominant role of toll-like receptor 2 versus 4 in Chlamydia pneumoniae-induced activation of dendritic cells.. J Immunol.

[37] Re F, Strominger J (2004). IL-10 released by concomitant TLR2 stimulation blocks the induction of a subset of Th1 cytokines that are specifically induced by TLR4 or TLR3 in human dendritic cells.. J Immunol.

[38] Belland R (2004). Chlamydia pneumoniae and atherosclerosis.. Cell Microbiol.

[39] Letiembre M, Echchannaoui H, Bachmann P, Ferracin F, Nieto C (2005). Toll-like receptor 2 deficiency delays pneumococcal phagocytosis and impairs oxidative killing by granulocytes.. Infect Immun.

[40] Shen Y, Kawamura I, Nomura T, Tsuchiya K, Hara H (2010). Toll-like receptor 2- and MyD88-dependent phosphatidylinositol 3-kinase and Rac1 activation facilitates the phagocytosis of Listeria monocytogenes by murine macrophages.. Infect Immun.

[41] Shin S, Behera A, Hu L (2008). The roles of MyD88, TLR2, TLR5, and TLR9 in phagocytosis and inflammatory signaling by Borrelia burgdorferi.. FASEB J.

[42] Luther K, Torosantucci A, Brakhage A, Heesemann J, Ebel F (2007). Phagocytosis of Aspergillus fumigatus conidia by murine macrophages involves recognition by the dectin-1 beta-glucan receptor and Toll-like receptor 2.. Cell Microbiol.

[43] Netea M, Sutmuller R, Hermann C, Van der Graaf C, Van der Meer J (2004). Toll-like receptor 2 suppresses immunity against Candida albicans through induction of IL-10 and regulatory T cells.. J Immunol.

[44] Thorburn A, O'Sullivan B, Thomas R, Kumar R, Foster P (2010). Pneumococcal conjugate vaccine-induced regulatory T cells suppress the development of allergic airways disease.. Thorax.

[45] Thorburn A, Hansbro P (2010). Harnessing regulatory T cells to suppress asthma: from potential to therapy.. Am J Respir Cell Mol Biol.

[46] Preston J, Thorburn A, Starkey M, Beckett E, Horvat J (2011). Streptococcus pneumoniae infection suppresses allergic airways disease by inducing regulatory T-cells.. Eur Respir J.

[47] Berry L, Hickey D, Skelding K, Bao S, Rendina A (2004). Transcutaneous immunization with combined cholera toxin and CpG adjuvant protects against Chlamydia muridarum genital tract infection.. Infect Immun.

[48] Skelding K, Hickey D, Horvat J, Bao S, Roberts K (2006). Comparison of intranasal and transcutaneous immunization for induction of protective immunity against Chlamydia muridarum respiratory tract infection.. Vaccine.

[49] Cheng C, Jain P, Bettahi I, Pal S, Tifrea D (2011). A TLR2 agonist is a more effective adjuvant for a Chlamydia major outer membrane protein vaccine than ligands to other TLR and NOD receptors.. Vaccine.

[50] Penttila J, Pyhala R, Sarvas M, Rautonen N (1998). Expansion of novel pulmonary CD3-, CD4+, CD8+ Cell Population in Mice during Chlamydia Pneumoniae Infection.. Infect Immun.

[51] Reutershan J, Morris M, Burcin T, Smith D, Chang D (2006). Critical role of endothelial CXCR2 in LPS-induced neutrophil migration into the lung.. J Clin Investig.

[52] Takenaka S, Safroneeva E, Xing Z, Gauldie J (2007). Dendritic cells derived from murine colonic mucosa have unique functional and phenotypic characteristics.. J Immunol.

[53] Oyoshi M, Barthel R, Tsitsikov E (2007). TRAF1 regulates recruitment of lymphocytes and, to a lesser extent, neutrophils, myeloid dendritic cells and monocytes to the lung airways following lipopolysaccharide inhalation.. Immunology.

[54] Feng H, Zhang D, Palliser D, Zhu P, Cai S (2005). Listeria-Infected myeloid dendritic cells produce IFN-, priming T Cell activation.. J Immunol.

[55] Preston J, Essilfie A, Horvat J, Wade M, Beagley K (2007). Inhibition of allergic airways disease by immunomodulatory therapy with whole killed Streptococcus pneumoniae.. Vaccine.

[56] Asquith K, Ramshaw H, Hansbro P, Beagley K, Lopez A (2008). The IL-3/IL-5/GM-CSF common receptor plays a pivotal role in the regulation of Th2 immunity and allergic airway inflammation.. J Immunol.

