# Supplementary figures and images for: TLR2, but Not TLR4, Is Required for Effective Host Defence against Chlamydia Respiratory Tract Infection in Early Life
Source: PLoS One. 2012 Jun 19;7(6):e39460. doi: 10.1371/journal.pone.0039460 (PMC3378543; doi:10.1371/journal.pone.0039460)

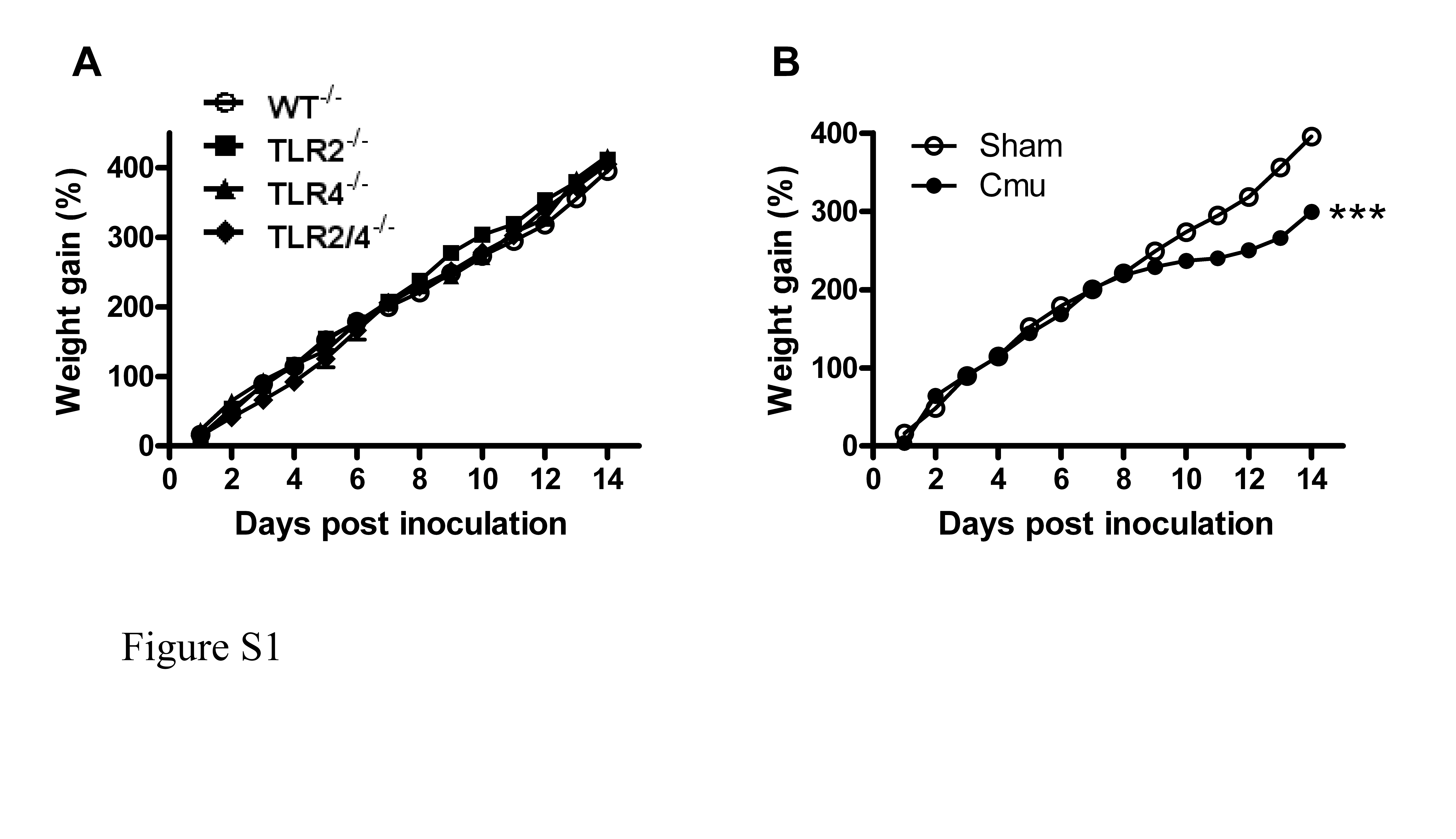

Supplement: Figure S1 — Absence of TLR2 does not affect growth in sham inoculated mice, but infection of Wt mice reduces weight gain. (A) Wild type (Wt) and TLR−/− pups were sham inoculated and weighed individually each day. The percentage weight gain was calculated daily relative to the average weight of the litter prior to inoculation. (B) Wt mice were sham inoculated or infected and weight gain monitored. n = 10–12 pups/group (5–6 pups/litter). Results are presented as means ± SEM. * denotes significant difference between sham inoculated and infected Wt groups. *** p<0.001 for the whole curves. (TIF) [file pone.0039460.s001.tif]

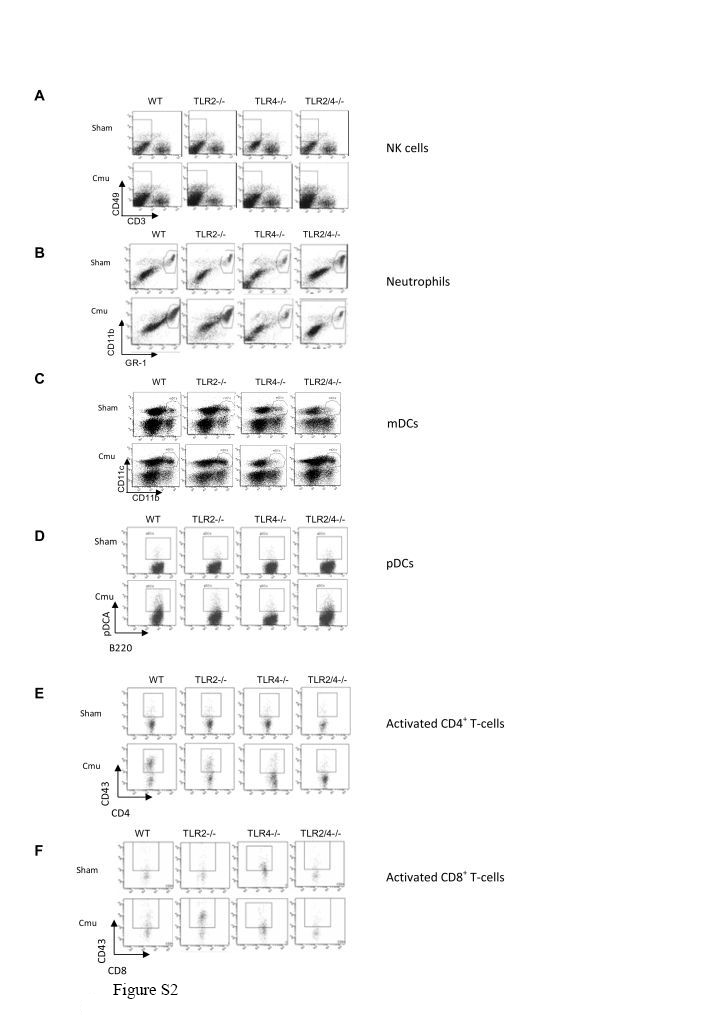

Supplement: Figure S2 — Absence of TLR2 increases NK and neutrophil influx and later DC and activated CD8+ T-cell recruitment during Chlamydia respiratory infection in early life. Representative FACS scatter plots of flow cytometric analysis of samples (A) NK Cells (B) Neutrophils (C) mDCs (D) pDCs (E) Activated CD4+ T-cells and (F) Activated CD8+ T-cells, as described in Figures 3–5, at 7 dpi. (TIF) [file pone.0039460.s002.tif]

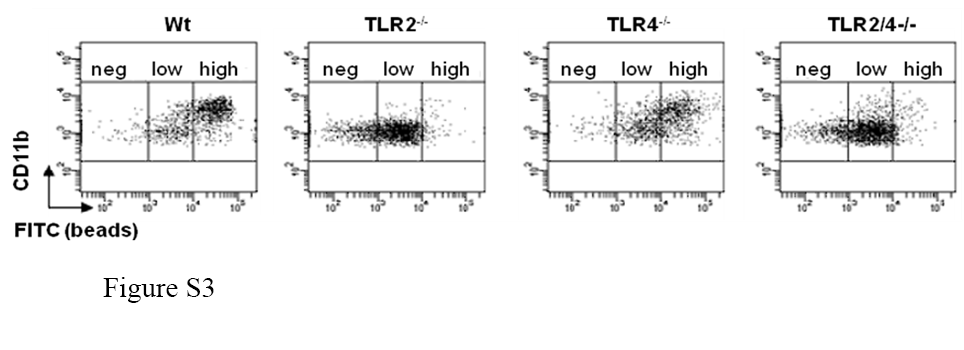

Supplement: Figure S3 — Absence of TLR2 reduces phagocytosis of Chlamydia by neutrophils. Representative FACS scatter plots of flow cytometric analysis of samples described in Figure 7. The numbers of low and high FITC-containing neutrophils were combined to produce the “Neutrophils FITC positive (%)” panel in Figure 7. (TIF) [file pone.0039460.s003.tif]

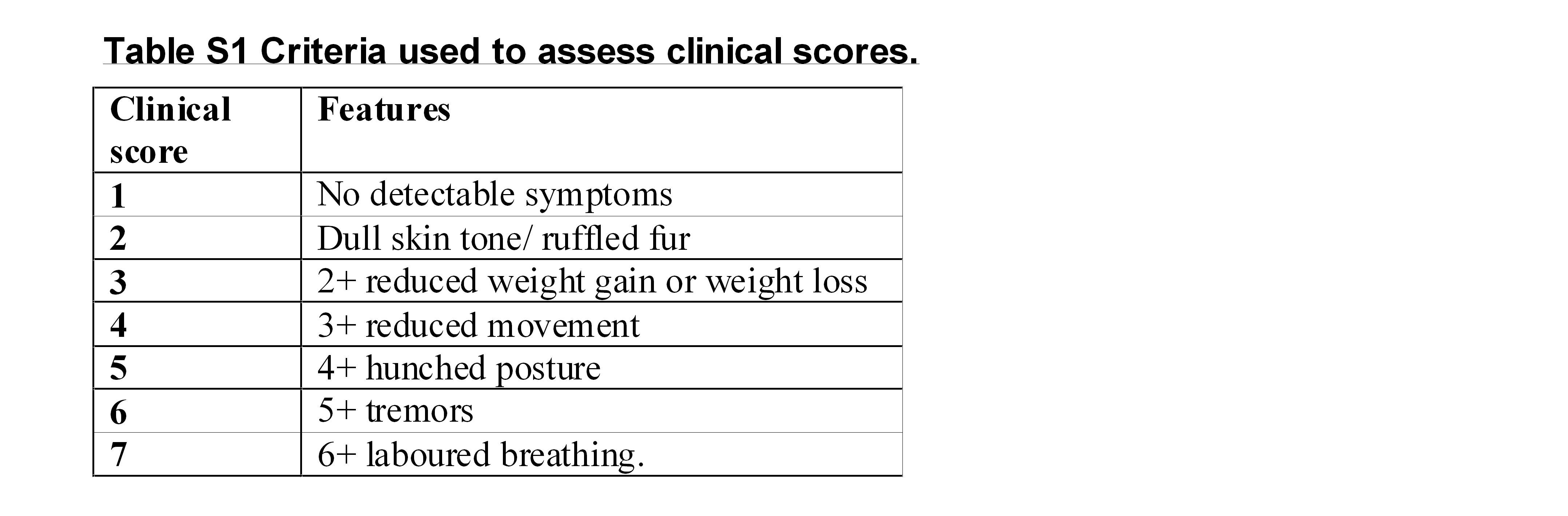

Supplement: Table S1 — Criteria used to assess clinical scores. (TIF) [file pone.0039460.s004.tif]

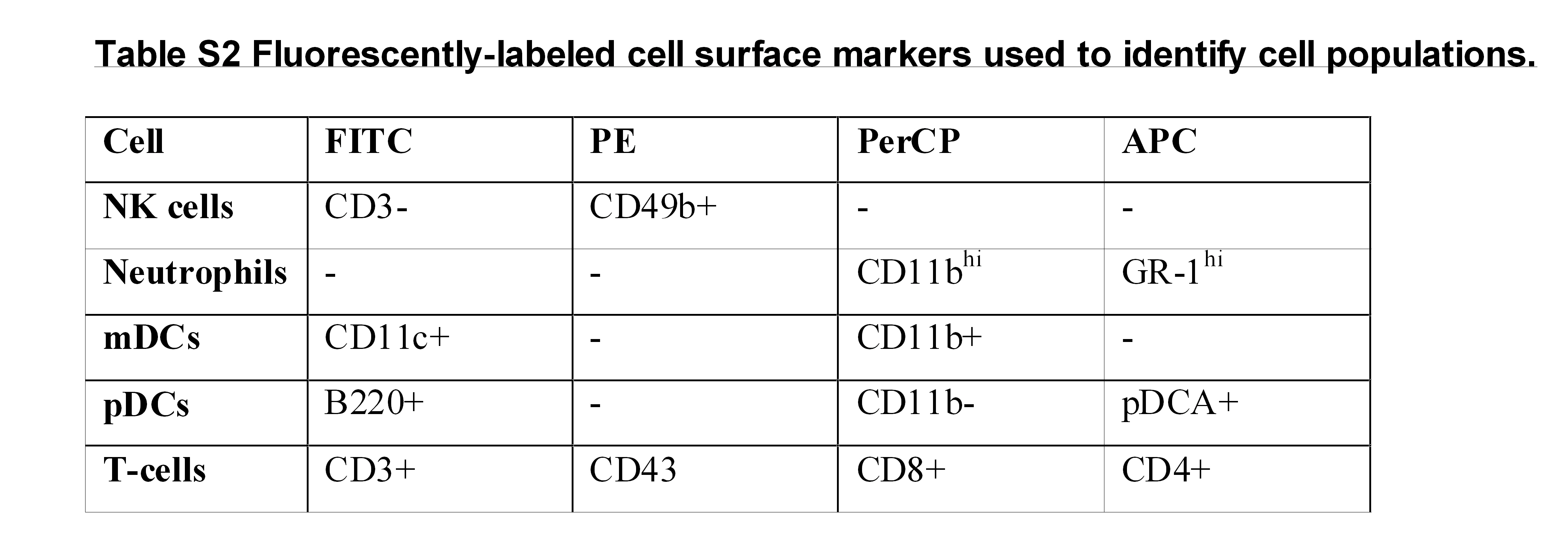

Supplement: Table S2 — Fluorescently-labeled cell surface markers used to identify cell populations. (TIF) [file pone.0039460.s005.tif]

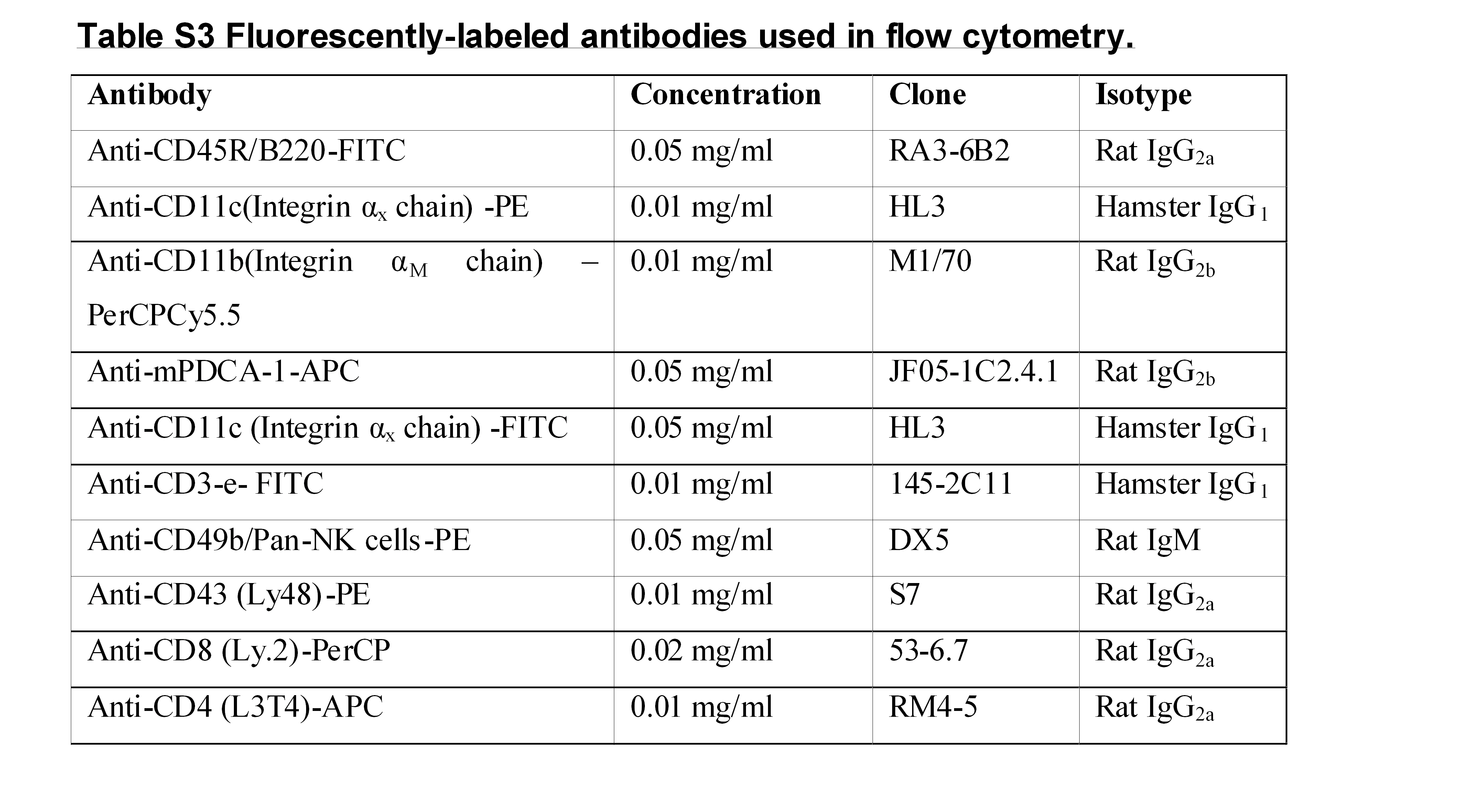

Supplement: Table S3 — Fluorescently-labeled antibodies used in flow cytometry. (TIF) [file pone.0039460.s006.tif]
